# Safety of darunavir/ritonavir (DRV/r) in HIV-1-infected DRV/r-experienced and -naïve patients: analysis of data in the real-world setting in Italy

**DOI:** 10.7448/IAS.17.4.19573

**Published:** 2014-11-02

**Authors:** Andrea Antinori, Marco Borderi, Roberto Cauda, Teresa Bini, Antonio Chirianni, Nicola Squillace, Daniela Mancusi, Roberta Termini

**Affiliations:** 1 Clinical Department, National Institute for Infectious Diseases “L Spallanzani”, Rome, Italy; 2 Department of Medical and Surgical Sciences, Infectious Disease Unit, Alma Mater Studiorum, University of Bologna, Bologna, Italy; 3 Institute of Infectious Diseases, A. Gemelli Hospital, Catholic University of the Sacred Heart, Rome, Italy; 4 Department of Medicine, Surgery and Dentistry, Clinic of Infectious Diseases, San Paolo Hospital, Milan, Italy; 5 Department of Infectious Disease, Cotugno Hospital, Naples, Italy; 6 Division of Infectious Diseases, Department of Internal Medicine, San Gerardo Hospital, Monza, Italy; 7 Janssen SpA, Medical Affairs, Cologno Monzese, Italy

## Abstract

**Introduction:**

This descriptive, non-interventional study on HIV-1-infected patients treated with DRV/r in the usual clinical setting, with a single-arm prospective observational design, collected data on utilization of darunavir/ritonavir (DRV/r) under the conditions described in marketing authorization in usual clinical practice in Italy to evaluate efficacy and safety of DRV/r-based antiretroviral (ARV) treatment. This analysis focussed on the safety profile of DRV/r in HIV-1 infected patients.

**Materials and Methods:**

Data were analyzed from four cohorts of HIV-1-infected patients treated with DRV/r in the real-world setting, including an ARV-naïve-DRV/r-naïve cohort (Cohort 1), an ARV-experienced-DRV/r-naïve cohort (Cohort 2) and two ARV-DRV/r-experienced cohorts (Cohorts 3 and 4), one of which (Cohort 3) was from the DRV/r Early Access Program. The objective of this analysis was to examine the safety data obtained in these four cohorts in patients enrolled from June 2009 to November 2011 and observed until December 2012 or DRV/r discontinuation.

**Results:**

Safety data from 875 patients were analyzed. DRV/r-based treatment was well tolerated, with 36.2% of patients reporting ≥1 adverse event (AE) and very few discontinuations due to study drug-related AEs (3.0% overall). The most frequent AEs were diarrhoea (2.7%), reduced bone density (2.6%) and hypercholesterolaemia (2.1%) (Table 1). Regarding metabolic parameters, levels of liver enzymes alanine aminotransferase (ALT) and aspartate aminotransferase (AST) remained stable from baseline to the last study visit (LSV) in DRV-experienced patients and decreased in DRV-naïve patients. Blood glucose concentrations remained stable in all cohorts. Serum triglyceride and cholesterol concentrations remained stable in DRV-experienced patients but increased in naïve patients, yet were still within normal range.

**Conclusions:**

In HIV-1-infected patients treated with DRV/r in these settings, the tolerability profile was favourable and similar to (or better than) that reported in controlled clinical trials. These data confirm DRV/r to be a safe treatment choice in DRV/r-experienced and naïve patients.

**Table 1:** Adverse events

AEs	All patients (N = 875); N (%)	Cohort 1 (N = 117); N (%)	Cohort 2 (N = 116); N (%)	Cohort 3 (N = 235); N (%)	Cohort 4 (N = 407); N (%)
One or more AEs	317 (36.2)	47 (40.2)	54 (46.6)	107 (45.5)	108 (26.5)
One or more ADRs	39 (4.5)	12 (10.3)	8 (6.9)	9 (3.8)	10 (2.5)
One or more serious AEs	105 (12.0)	19 (16.2)	16 (13.8)	26 (11.1)	43 (10.6)
Death	26 (3.0)	3 (2.6)	4 (3.4)	10 (4.3)	9 (2.2)
Darunavir treatment stopped because of AEs	26 (3.0)	8 (6.8)	6 (5.2)	3 (1.3)	9 (2.2)
AE Description					
Rash	10 (1.1)	4 (3.4)	4 (3.4)	2 (0.9)	0
Diarrhoea	24 (2.7)	3 (2.6)	4 (3.4)	8 (3.4)	9 (2.2)
Hypertension	15 (1.7)	1 (0.9)	2 (1.7)	10 (4.3)	2 (0.5)
Hepatic enzymes increased	10 (1.1)	4 (3.4)	1 (0.9)	3 (1.3)	2 (0.5)
Hypercholesterolaemia	18 (2.1)	2 (1.7)	2 (1.7)	8 (3.4)	6 (1.5)
Hypertriglyceridemia	15 (1.7)	1 (0.9)	3 (2.5)	6 (2.6)	5 (1.2)
Hyperlipaemia	11 (1.3)	5 (4.3)	1 (0.9)	2 (0.9)	3 (0.7)
Reduced Bone Mineral Density	23 (2.6)	1 (0.9)	3 (2.6)	10 (4.3)	9 (2.2)
Fever	14 (1.6)	6 (5.1)	0	5 (2.1)	3 (0.7)

